# The order of infection shapes disease outcomes in influenza and herpes simplex virus coinfection by modulating immune responses

**DOI:** 10.1186/s12985-025-02968-4

**Published:** 2025-10-31

**Authors:** Yuanjun Lyu, Kailin Mai, Hongxuan Zhou, Chunguang Yang, Yunceng Weng, Zhenhui Zhang, Yang Wang, Zifeng Yang

**Affiliations:** 1 National Clinical Research Center for Respiratory Disease, State Key Laboratory of Respiratory Disease, Guangzhou Institute of Respiratory Health, the First Affiliated Hospital of Guangzhou Medical University, Guangzhou, 510120 China; 2 Henan University College of Medicine, Kaifeng, 475004 China; 3 Department of Critical Care, The Second Affiliated Hospital of Guangzhou Medical University, Guangzhou, 510260 China; 4 Department of Respiratory Medicine, The First Affiliated Hospital of Zhengzhou University, Zhengzhou, 450052 China; 5 Guangzhou National Laboratory, Guangzhou, 510005 China; 6 State Key Laboratory of Quality Research in Chinese Medicine, Macau University of Science and Technology, Macau (SAR), Taipa, 519020 China; 7 Respiratory Disease AI Laboratory on Epidemic and Medical Big Data Instrument Applications, Faculty of Innovation Engineering, Macau University of Science and Technology, Macau (SAR), Taipa, 519020 China

**Keywords:** Influenza a virus, Herpes simplex virus type 1, Coinfection, Immune response

## Abstract

**Background:**

Coinfections of influenza A virus (IAV) and herpes simplex virus type 1 (HSV-1) have been increasingly reported in patients with severe pneumonia, yet their pathogenesis remains poorly understood.

**Methods:**

We established murine models to investigate the effects of coinfection with HSV-1 (strain KOS) and IAV (A/Aichi/2/1968 (H3N2)) under different orders of infection. Mice were assigned to one of five groups: (1) HSV-1 monoinfection, (2) H3N2 monoinfection, (3) simultaneous coinfection (H3N2 + HSV-1), (4) sequential coinfection with H3N2 administered three days prior to HSV-1 (H3N2-HSV-1), and (5) sequential coinfection with HSV-1 administered three days prior to H3N2 (HSV-1-H3N2). We then compared disease severity, viral replication, lung injury, cytokine profiles along with innate and adaptive immune responses.

**Results:**

Overall, all coinfection groups developed more severe disease than HSV-1 monoinfection. However, when compared to H3N2 monoinfection, the order of coinfection resulted in distinct differences in disease severity and immune response patterns. Specifically, simultaneous H3N2 + HSV-1 and sequential H3N2-HSV-1 coinfections led to increased mortality, higher H3N2 viral loads, and more pronounced pulmonary inflammation. These groups exhibited elevated cytokine levels and dysregulated immune responses, with the H3N2 + HSV-1 group displaying reduced proportions of natural killer cells (NK), plasmacytoid dendritic cells (pDCs) and interferon-producing killer dendritic cells (IKDCs) in bronchoalveolar lavage fluid (BALF), while the H3N2-HSV-1 group showed a robust expansion of these innate immune cells. Additionally, these two coinfection strategies were associated with increased H3N2-specific IFN-γ⁺CD8⁺ T cells, reflecting an exacerbated adaptive response. In contrast, sequential HSV-1-H3N2 coinfection resulted in milder disease manifestations, characterized by lower mortality, decreased clinical severity, reduced cytokine levels, and diminished proportions of NK cells, pDCs, IKDCs, and CD8⁺ T cells in BALF. Moreover, H3N2-specific IFN-γ⁺CD8⁺ T cells were reduced in both lung and spleen tissues, indicating a more controlled immune activation during HSV-1-H3N2 coinfection.

**Conclusion:**

Our study demonstrates that the infection order of H3N2 and HSV-1 coinfection critically shapes disease outcomes. Specifically, sequential H3N2 infection preceding HSV-1 or simultaneous coinfection with H3N2 and HSV-1 exacerbated immunopathology. Conversely, prior HSV-1 exposure attenuated H3N2-driven inflammation via reduced cytokine levels and immune cell recruitment. This study provides novel insights into immune dysregulation in coinfection models, with potential translational implications for managing influenza and herpesvirus coinfections.

## Introduction

With advancements in diagnostic technologies, viral coinfections are increasingly identified in critically ill patients, particularly those with severe pneumonia [1–3]. As one of the most prevalent respiratory viruses, influenza A virus (IAV) is a common causative pathogen of severe pneumonia, contributing significantly to global mortality from respiratory diseases [4]. Studies indicate that IAV frequently occurs in respiratory viral coinfections, with incidence of coinfection ranging from 4.7 to 20% [4, 5]. However, the impact of IAV coinfections with other respiratory viruses on disease severity remains debated [6]. During the 2009 H1N1 pandemic, coinfection with rhinovirus was associated with lower disease severity and more rapid recovery in patients infected with IAV, suggesting a potential protective effect [5]. In contrast, coinfection with IAV and respiratory syncytial virus (RSV) was linked to worse clinical outcomes, potentially due to immune evasion through the formation of hybrid virus particles that circumvent anti-IAV neutralizing antibodies [7, 8].

Meanwhile, herpes simplex virus type 1 (HSV-1) is frequently detected in patients requiring mechanical ventilation for severe pneumonia [9, 10]. Typically, HSV-1 is neurotropic and establishes latency in ganglia. The presence of HSV-1 in the lower respiratory tract is often considered a consequence of reactivation caused by immune dysregulation [11]. The increased detection of HSV-1 in severe pneumonia patients is primarily due to an immunosuppressive state characterized by T cell and natural killer cell exhaustion [12]. During the COVID-19 pandemic, the high incidence of HSV-1 coinfection among severe COVID-19 patients has drawn attention, yet a comparative study showed that the incidence of HSV lung reactivation was higher in influenza patients than in COVID-19 patients [13]. However, despite its increasing prevalence, the impact of HSV-1 coinfections on clinical outcomes remains controversial. Our previous study demonstrated that co-infection with avian influenza virus (H7N9 subtype) and HSV-1 led to rapid disease progression, and another report showed that coinfection with seasonal influenza virus and HSV-1 caused extensive lung consolidation [14, 15]. In contrast, a study reported that HSV lung reactivation had no impact on the outcomes of influenza or COVID-19 patients [13]. Furthermore, in mechanically ventilated patients with HSV coinfection, the efficacy of anti-HSV treatment in alleviating lung injury remains uncertain [16]. These inconclusive findings suggest that, during coinfection with HSV-1 and respiratory viruses, HSV-1 may contribute to disease severity primarily by modulating the host immune response.

In this study, we aimed to investigate the pathogenesis of IAV and HSV-1 coinfection by comparing disease severity and immune responses induced by different orders of infection. By elucidating the impact of coinfection on disease outcomes, this study provides insights into the mechanisms and clinical management of viral coinfections.

## Materials and methods

### Cell lines

Madin-Darby canine kidney cells (MDCK; ATCC#CCL-34) and vero cells (ATCC#CCL-81) were used. Cells were grown in Dulbecco’s Modified Eagle’s Medium/Nutrient Mixture F-12 (DMEM/F12, Gibco) with 10% FBS, 1% antibiotics (penicillin 100 U/ml and streptomycin 100 mg/ml) at 37 °C with 5% CO_2_.

### Animals

Six to eight-week-old female C57BL/6 mice were obtained from Experimental Animal Center of Guangdong Province. All mice were housed under specific pathogen-free (SPF) conditions. Animal experiments were approved by the Animal Care and Use Committee of the First Affiliated Hospital of Guangzhou Medical University (No. 20240470).

### Viruses and infection

Mouse-adapted influenza virus A/Aichi/2/1968(H3N2) obtained from ATCC was propagated in 10-day-old embryonated chicken eggs. HSV-1 KOS strain was grown and titrated in vero cells (ATCC#CCL-81). All virus stocks were stored at −80 °C until required.

Mice were anesthetized with isoflurane, and inoculated intranasally with viruses diluted in PBS or PBS with total volume of 50µL. For the HSV-1-H3N2 group, mice were infected with 10^4^ PFU HSV-1, and sequentially infected with 200 PFU H3N2 after three days. For the H3N2 + HSV-1 group, mice were simultaneously infected with 200 PFU H3N2 and 10^4^ PFU HSV-1. For the H3N2-HSV-1 group, mice were infected with 10 PFU H3N2, and sequentially infected with10^4^ PFU HSV-1 after three days. Control groups included mock controls and monoinfection controls, which were given either H3N2 or HSV-1 monoinfection at the corresponding time points and the corresponding infection doses. The body weight of mice was monitored daily, and mice with weight loss above 30% of original weight were euthanized and recorded as dead. For consistency, the day of H3N2 infection was designated as 0 days post infection (0 dpi) in three groups.

### Lung index and histopathology

At 4, 6, 8, and 10 dpi, lungs were collected from mice and weighed. The lung index was calculated as 100% × lung weight/body weight. Additionally, at 8 dpi, lungs were collected and fixed with 10% formalin for 24 h. The lung tissues were then embedded in paraffin, sectioned and stained with hematoxylin and eosin (H&E).

### Bronchoalveolar lavage fluid and lung homogenate Preparation

To collect bronchoalveolar lavage fluid (BALF), mice were sacrificed and lungs were lavaged three times with cold PBS via blunted needles inserted into tracheas. For flow cytometry, BALF were kept on ice before staining. For cytokine detection, the fluids were then centrifugated at 1000 rpm at 4℃ for 10 min, and supernatants were collected for subsequent analysis. To prepare lung homogenates, lungs were collected and homogenized in PBS, then centrifugated at 12,000 rpm at 4℃ for 10 min, and supernatants were collected.

### Virus titration

Foci assay was used for virus titration. Supernatants of lung homogenates at 4, 6, 8, and 10 dpi were serially diluted 10-fold and inoculated into MDCK or vero cells at 37 °C with 5% CO_2_ for 60 min and each well was overlayed with 1.6% carboxymethylcellulose for two days. Then cells were fixed with 4% paraformaldehyde for 20 min and 0.5% Triton X-100 for 10 min. Anti-nucleoprotein (anti-NP) antibody (Novus, dilution 1:4000) or anti-HSV-1 antibody (Abcam, dilution 1:2500) were added and incubated for 60 min. After washing with PBST for three times, each well was given anti-mouse-IgG-HRP antibody or anti-rabbit-IgG-HRP antibody. AEC kit (Sigma- Aldrich) was used for staining.

### Measurement of cytokine levels

At 8 dpi, BALF were collected for cytokine measurement. Concentrations of IFN-γ, TNF-α, IP-10, MCP-1, IL-10, and IL-6 were detected by Mouse Cytokine 6-Plex Antibody Bead Kit (Invitrogen) on Luminex™ 100/200 Magpix™ Analyzer. Concentrations were calculated from standard curves in unit of pg/mL.

### Preparing single-cell suspensions for flow cytometry

For innate immune cell analysis, BALF were collected at 6 and 8 dpi. For T cell subset analysis, BALF and spleens were collected at 6 dpi. For NP-specific CD8^+^ T cell analysis, lungs and spleens were collected at 10 dpi. To prepare single-cell suspensions, lung tissues were minced before incubation with collagenase D (Roche, 1 mg/mL) and DNase (Roche, 10 U/mL) at 37℃ for 30 min. The digested lung tissues and spleen were then mechanically dissociated by passage through 70 μm nylon cell strainer and centrifuged before lysing erythrocytes. Cells were then washed twice and resuspended in PBS.

### Flow cytometric analysis

For surface marker staining, single-cell suspensions of BALF and splenocytes were incubated with 1:200 dilution of anti-CD16/32 (BD Bioscience) on ice for 30 min. The cells were then labeled with 1:200 dilution of cell surface antibodies: anti-mouse CD3 (APC, eBioscience), anti-mouse CD8a (BV421, BioLegend), anti-mouse CD4 (PerCP/cy5.5, BioLegend), anti-mouse CD3 (APC/FireTM750, BioLegend), anti-mouse B220 (PE, BioLegend), anti-mouse NK1.1 (BV421, BioLegend), anti-mouse CD11c (BV421, BioLegend), anti-mouse F4/80 (Alexa Fluor^®^647, BioLegend) and anti-mouse/human CD11b (FITC, BioLegend). After staining on ice for 30 min, cells were washed twice and fixed in 4% paraformaldehyde. Cells were then washed, resuspended in PBS and analyzed. Natural killer (NK) cells were identified as CD3^−^NK1.1^+^ cells. Plasmacytoid dendritic cells (pDC) were identified as CD3^−^B220^+^CD11c^+^ cells. Interferon-producing killer dendritic cells (IKDC) were identified as CD3^−^B220^+^NK1.1^+^CD11c^+^ cells. Macrophages were identified as CD11b^+^F4/80^+^ cells. CD4^+^ T cells were identified as CD3^+^CD4^+^CD8^−^ cells. CD8^+^ T cells were identified as CD3^+^CD4^−^CD8^+^ cells.

To analyze NP-specific CD8^+^ T cells, single-cell suspensions of lungs and spleens were prepared as described above. Influenza A (H3N2 subtype) peptides NP_366−374_ (ASNENMETM) were synthesized by Kangbei Biotechnology. Next, 10^6^ cells per well were seeded in 96-well U-shape plates and stimulated with 1 µM of influenza NP_366−374_ peptide in the presence of Golgi Plug (BD Biosciences, 1 µL/mL) for 6 h at 37℃. After washing twice, cells were blocked and stained with Live/Dead-aqua 525 (Invitrogen, 1:400) and labelled with 1:200 dilution of surface marker antibodies: anti-mouse CD3 (APC/Fire^TM^750, BioLegend), anti-mouse CD8a (PerCP-Cyanine5.5, BioLegend). After washing and fixation/permeabilization (BD Biosciences) for 20 min, cells were intracellularly stained with 1:200 dilution of anti-mouse IFN-γ (BV421, BioLegend). Finally, cells were resuspended in PBS and analyzed. NP-specific CD8^+^ T cells were identified as CD8^+^ T cells with IFN-γ secretion after NP_366−374_ stimulation (IFN-γ^+^CD8^+^ T cells).

### Statistical analysis

Data are presented as mean ± SD. Immune cells were gated and analyzed using FlowJo software (v10.8). Statistical analyses were conducted in GraphPad Prism (v5.0). For comparisons between two groups, a two-tailed Student’s t-test was applied when variances were equal. When variances were unequal, an unpaired Mann-Whitney test was used. Survival rate differences were assessed using the log-rank test. Statistical significance was defined as *P* < 0.05. *, *P* < 0.05; **, *P* < 0.01; ***, *P* < 0.001.

## Results

### Coinfection timing drives divergent disease severity and influenza viral replication

**Fig. 1:**
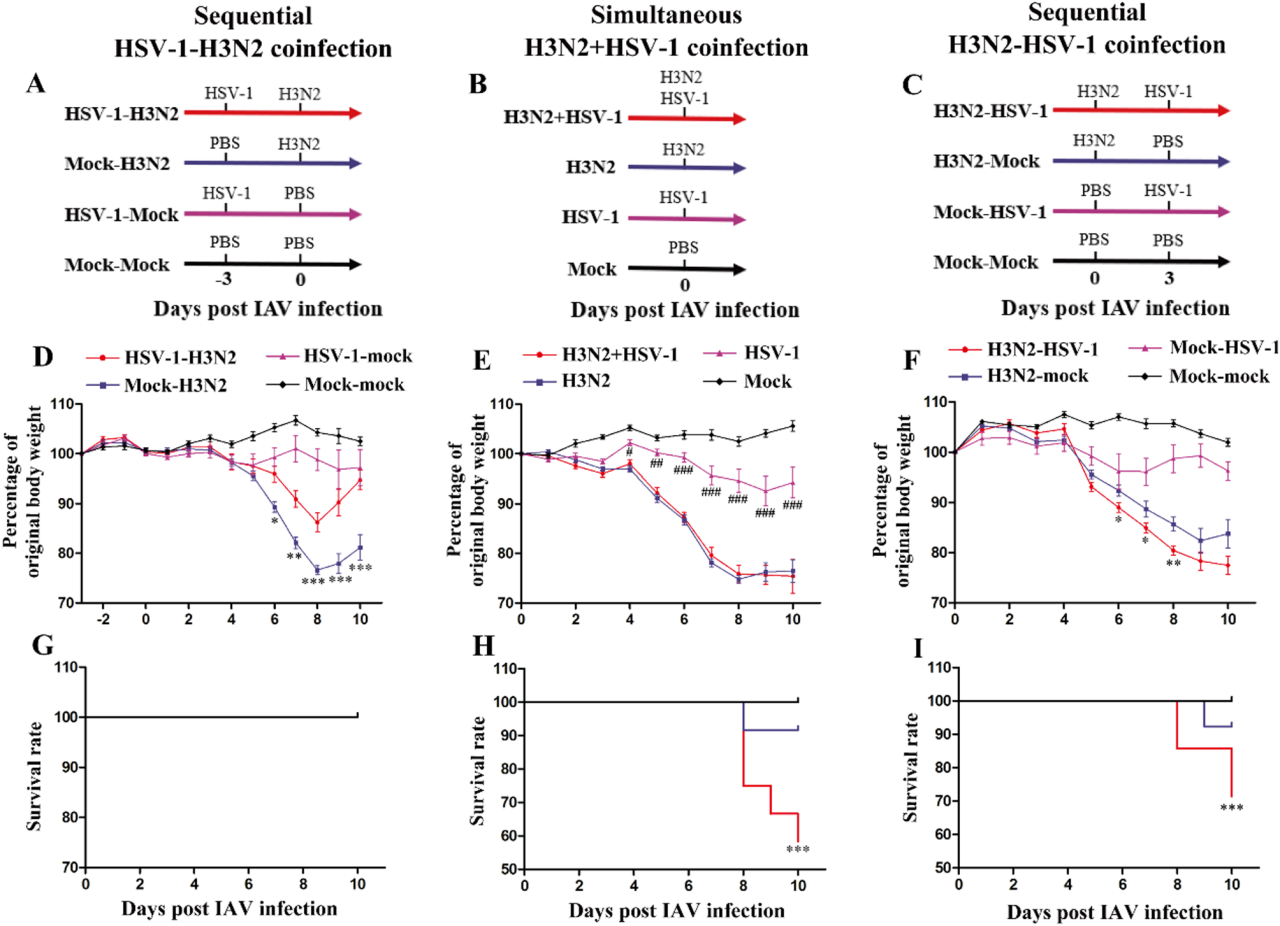
Weight change and survival rate in three groups of H3N2 and HSV-1 coinfection models. (**A-C**) Schemes for three groups of coinfection models with different orders of infection. Daily weight change (**D-F**) and survival rate (**G-I**) in different coinfection models. Sequential HSV-1-H3N2 coinfection group: (**A**), (**D**), (**G**); simultaneous H3N2 + HSV-1 coinfection group: (**B**), (**E**), (**H**); sequential H3N2-HSV-1 coinfection group: (**C**), (**F**), (**I**). Data are shown as mean ± SD. Significant differences compared to H3N2 monoinfection controls are marked by asterisk (^*^), and to HSV-1 monoinfection controls by hash (^#^). (^*/#^, *P* < 0.05; ^**/##^, *P* < 0.01; ^***/###^, *P* < 0.001)

**Fig. 2:**
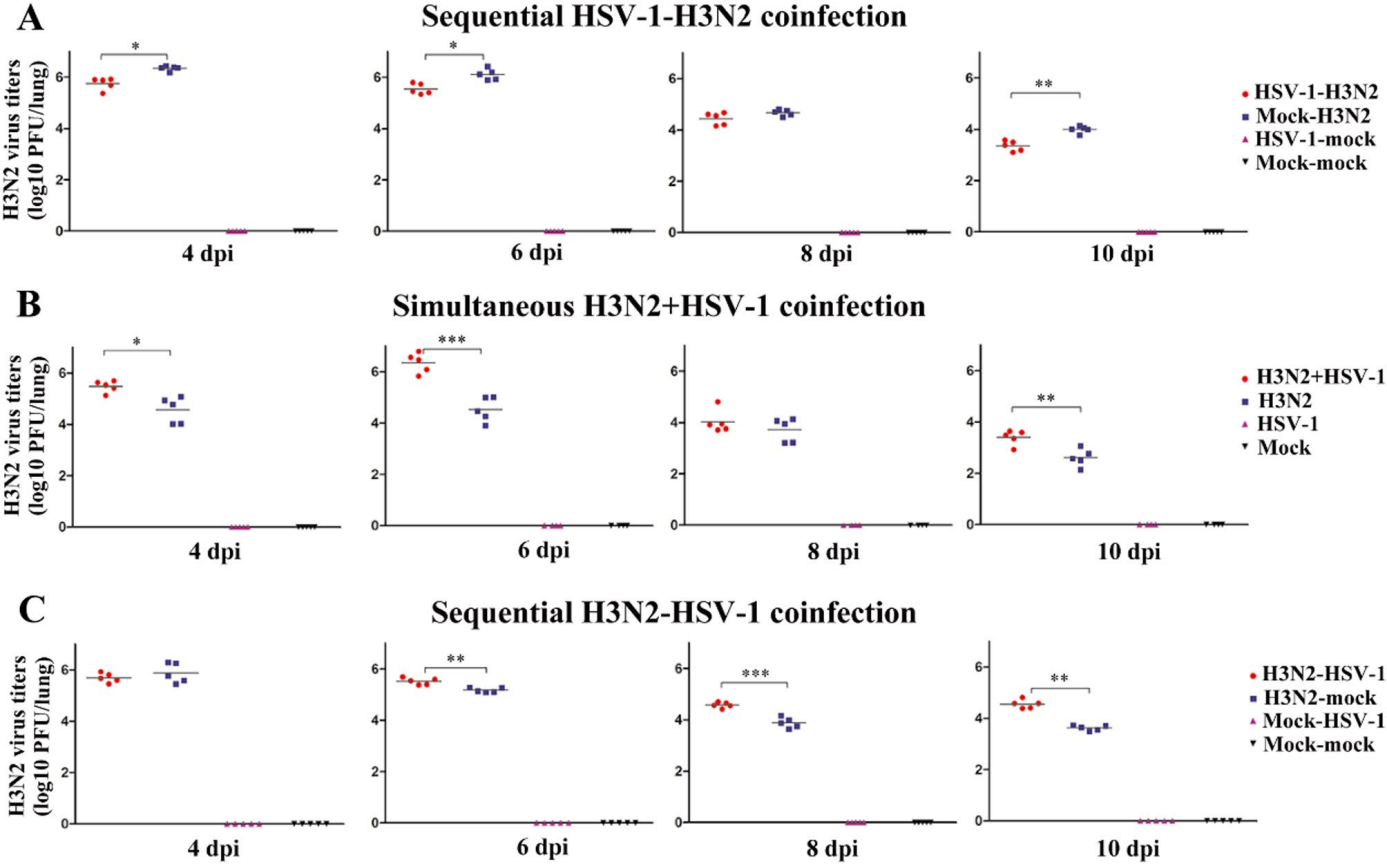
H3N2 titers in the lungs of coinfection groups. (**A-C**) H3N2 virus titers were measured in the lungs at 4, 6, 8, and 10 days post-H3N2 infection (dpi) in the sequential HSV-1-H3N2 group (**A**), simultaneous H3N2 + HSV-1 group (**B**), and sequential H3N2-HSV-1 group (**C**). Data are presented as individual points with the mean. Significant differences between coinfection models and H3N2 monoinfection controls are marked by asterisk (^*^). (^*^, *P* < 0.05; ^**^, *P* < 0.01; ^***^, *P* < 0.001)

**Fig. 3:**
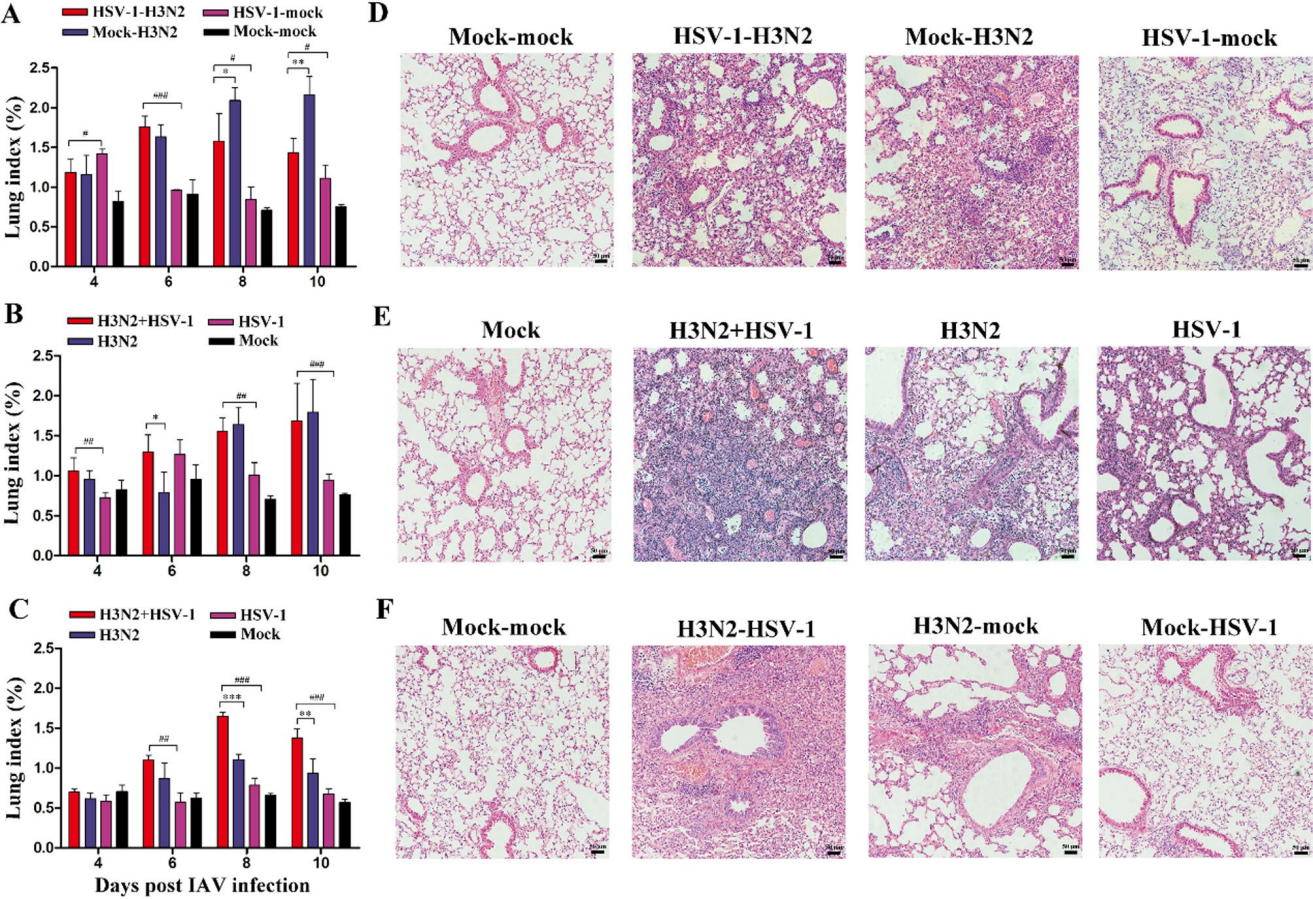
Lung index and histopathological analysis of coinfection groups. (**A-C**) Lung index at 4, 6, 8, and 10 dpi were measured in the sequential HSV-1-H3N2 group (**A**), simultaneous H3N2 + HSV-1 group (**B**) and sequential H3N2-HSV-1 group (**C**). Data are shown as mean ± SD. Significant differences between coinfection models and H3N2 monoinfection controls are marked by asterisk (^*^), and differences between coinfection models and HSV-1 monoinfection controls by hash (^#^). (^*/#^, *P* < 0.05; ^**/##^, *P* < 0.01; ^***/###^, *P* < 0.001) (**D-F**) Histopathological analysis of the lungs at 8 dpi was performed in the sequential HSV-1-H3N2 group (**D**), simultaneous H3N2 + HSV-1 group (**E**), and sequential H3N2-HSV-1 group (**F**). Scale bar: 50 μm

To compare disease severity across different coinfection timing, we established three coinfection models: (1) sequential HSV-1-H3N2 (Fig. 1A), (2) simultaneous H3N2 + HSV-1 (Fig. 1B), and (3) sequential H3N2-HSV-1 (Fig. 1C). Each model included corresponding mock controls that received PBS or monoinfection of either H3N2 or HSV-1 at the relevant time points. For consistency, the day of H3N2 infection was designated as day 0 post infection (0 dpi).

Compared to the mock-H3N2 group, the HSV-1-H3N2 group showed alleviated weight loss from 6 to 10 dpi (Fig. 1D). Meanwhile, the H3N2 + HSV-1 group showed a comparable pattern of weight loss to the H3N2 group (Fig. 1E). Likewise, the H3N2-HSV-1 group showed a comparable pattern and exhibited even greater weight loss between 6 and 8 dpi relative to the H3N2-mock group (Fig. 1F). Coinfected with 200 PFU of H3N2 virus, the HSV-1-H3N2 group (Fig. 1G) exhibited no mortality (100% survival rate). In contrast, the H3N2 + HSV-1 group (Fig. 1H), infected with the same dose of H3N2, showed a significantly higher mortality rate compared to the H3N2 group (41% vs. 8.5%, *P* < 0.001). Similarly, the H3N2-HSV-1 group (Fig. 1I), coinfected with a lower dose of H3N2 (10 PFU), also demonstrated a higher mortality rate than the H3N2-mock group (28.5% vs. 7.6%, *P* < 0.001). Of note, HSV-1 monoinfection controls did not cause significant weight loss or mortality. Compared to HSV-1 monoinfection controls, H3N2 coinfection resulted in greater weight loss and mortality, irrespective of the order of infection.

To investigate the impact of HSV-1 on H3N2 viral replication in different coinfection groups, H3N2 virus titers in the lungs were measured at 4, 6, 8, and 10 dpi. Compared to the mock-H3N2 group, the HSV-1-H3N2 group exhibited reduced H3N2 viral titers at 4, 6, and 10 dpi (Fig. 2A). In contrast, the H3N2 + HSV-1 group showed higher H3N2 titers than the H3N2 group at 4, 6, and 10 dpi (Fig. 2B). Similarly, the H3N2-HSV-1 group displayed increased H3N2 titers compared to the H3N2-mock group at 6, 8, and 10 dpi (Fig. 2C). These results suggest that prior HSV-1 infection could accelerate the clearance of H3N2 virus and mitigate influenza-induced lethality. On the other hand, simultaneous coinfection and sequential infection with H3N2 prior to HSV-1 could enhance H3N2 viral replication and lead to more severe disease.

### Simultaneous and H3N2-preceding coinfection exacerbate lung injury and inflammation

To assess the severity of lung injury in three groups of coinfection models, lung index was calculated at 4, 6, 8, and 10 dpi as an indicator of pulmonary edema and inflammation. The HSV-1-H3N2 group had reduced lung index at 8 and 10 dpi compared to mock-H3N2 group (Fig. 3A), whereas the H3N2 + HSV-1 group showed increased lung index at 6 dpi compared to H3N2 group (Fig. 3B). Likewise, the lung index of H3N2-HSV-1 group was higher than the H3N2-mock group at 8 and 10 dpi (Fig. 3C). Additionally, compared to HSV-1 monoinfection controls, all three groups with H3N2 and HSV-1 coinfection resulted in higher lung indexes.

Lungs were collected at 8 dpi for histopathological analysis to assess lung injury. Interstitial lesions, a hallmark of viral pneumonia [17], were significantly present in all three coinfection models and the H3N2 monoinfection controls. Notably, compared to the mock-H3N2 group, the HSV-1-H3N2 group showed reduced inflammatory cell infiltration (Fig. 3D). In contrast, compared to H3N2 monoinfection controls, H3N2 + HSV-1 coinfection caused more pronounced thickening of alveolar septa and increased inflammatory infiltration (Fig. 3E), with similar lesions observed in the H3N2-HSV-1 group (Fig. 3F). Of note, all three H3N2 and HSV-1 coinfection models exhibited more severe lung injury than HSV-1 monoinfection controls.

**Fig. 4:**
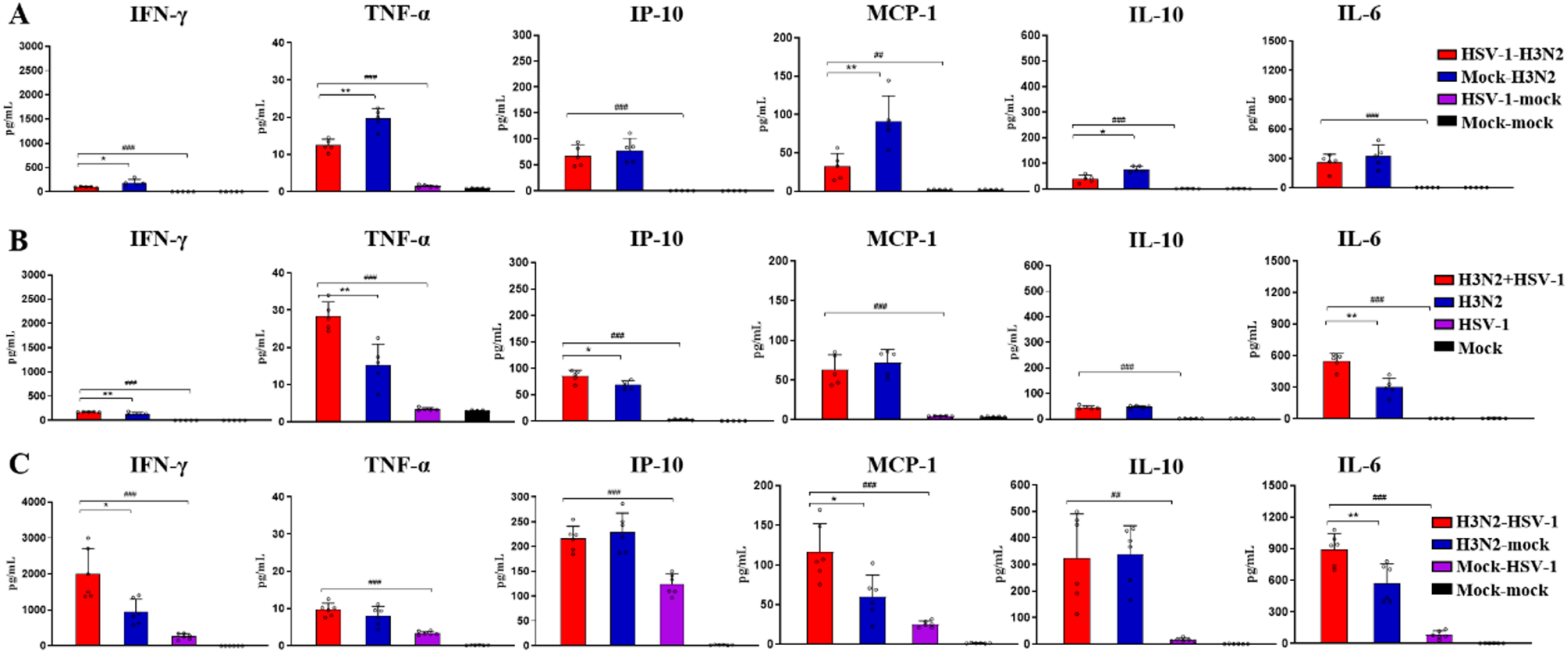
BALF cytokine levels in coinfection groups. (**A-C**) BALF concentrations of IFN-γ, TNF-α, IP-10, MCP-1, IL-10, and IL-6 were measured at 8 dpi in the sequential HSV-1-H3N2 group (**A**), simultaneous H3N2 + HSV-1 group (**B**) and sequential H3N2-HSV-1 group (**C**). Data are shown as mean ± SD. Significant differences between coinfection models and H3N2 monoinfection controls are marked by asterisk (^*^), and between coinfection models and HSV-1 monoinfection controls by hash (^#^). (^*/#^, *P* < 0.05; ^**/##^, *P* < 0.01; ^***/###^, *P* < 0.001)

To further assess pulmonary inflammation, cytokine levels were measured in BALF at 8 dpi. Compared to the H3N2 monoinfection control, the HSV-1-H3N2 group exhibited reduced levels of IFN-γ, TNF-α, MCP-1, and IL-10 (Fig. 4A). In contrast, the H3N2 + HSV-1 group showed increased levels of IFN-γ, TNF-α, IP-10, and IL-6 (Fig. 4B), and the H3N2-HSV-1 group exhibited elevated levels of IFN-γ, MCP-1, and IL-6 (Fig. 4C). Although the lung index showed no significant differences between the H3N2 + HSV-1 and H3N2 groups at 8 to 10 dpi (Fig. 3B), elevated levels of BALF cytokines of the H3N2 + HSV-1 group compared to H3N2 group at 8 dpi indicated more severe pulmonary inflammation despite similar lung edema. This is consistent with the higher mortality observed in the H3N2 + HSV-1 group. These results demonstrated that simultaneous H3N2 + HSV-1 and sequential H3N2-HSV-1 coinfections exacerbated lung injury and inflammation compared to H3N2 monoinfection, whereas sequential HSV-1-H3N2 coinfection mitigated these effects.

### Simultaneous and sequential coinfection drive distinct immune cell profiles

**Fig. 5:**
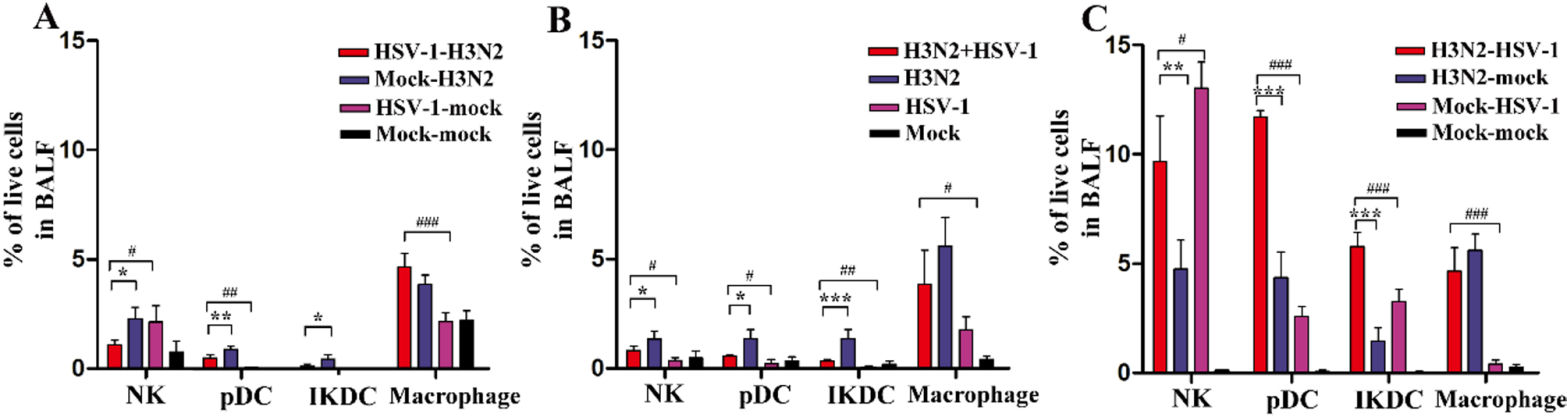
Innate immune cell profiles in different coinfection groups. (**A-C**) Proportions of NK, pDC, IKDC and macrophages in BALF were measured at 8 dpi in the sequential HSV-1-H3N2 group (**A**), simultaneous H3N2 + HSV-1 group (**B**), and sequential H3N2-HSV-1 group (**C**). Data are shown as mean ± SD. Significant differences between coinfection models and H3N2 monoinfection controls are marked by asterisk (^*^), and between coinfection models and HSV-1 monoinfection controls by hash (^#^). (^*/#^, *P* < 0.05; ^**/##^, *P* < 0.01; ^***/###^, *P* < 0.001)

Since histopathological and cytokine analyses indicated that inflammation significantly contributed to the pathogenesis of H3N2 and HSV-1 coinfection, we hypothesized that altered immune cell profiles could be linked to this inflammation. First, we evaluated innate immune cells involved in antiviral and pro-inflammatory responses, including NK cells, pDCs, IKDCs, and macrophages, in BALF at 8 dpi. Compared to the mock-H3N2 group, the proportions of NK cells, pDCs, and IKDCs were significantly reduced in the HSV-1-H3N2 group (Fig. 5A). A similar trend was observed in the H3N2 + HSV-1 group compared to the H3N2 group (Fig. 5B). In contrast, the H3N2-HSV-1 group exhibited a significant increase in NK cells, pDCs, and IKDCs compared to the H3N2-mock group (Fig. 5C). Compared to HSV-1 monoinfection controls, all three coinfection models showed elevated proportions of pDCs, IKDCs, and macrophages. The level of NK cells was higher in the H3N2 + HSV-1 group, but lower in the HSV-1-H3N2 and H3N2-HSV-1 groups than in HSV-1 monoinfection controls.

**Fig. 6:**
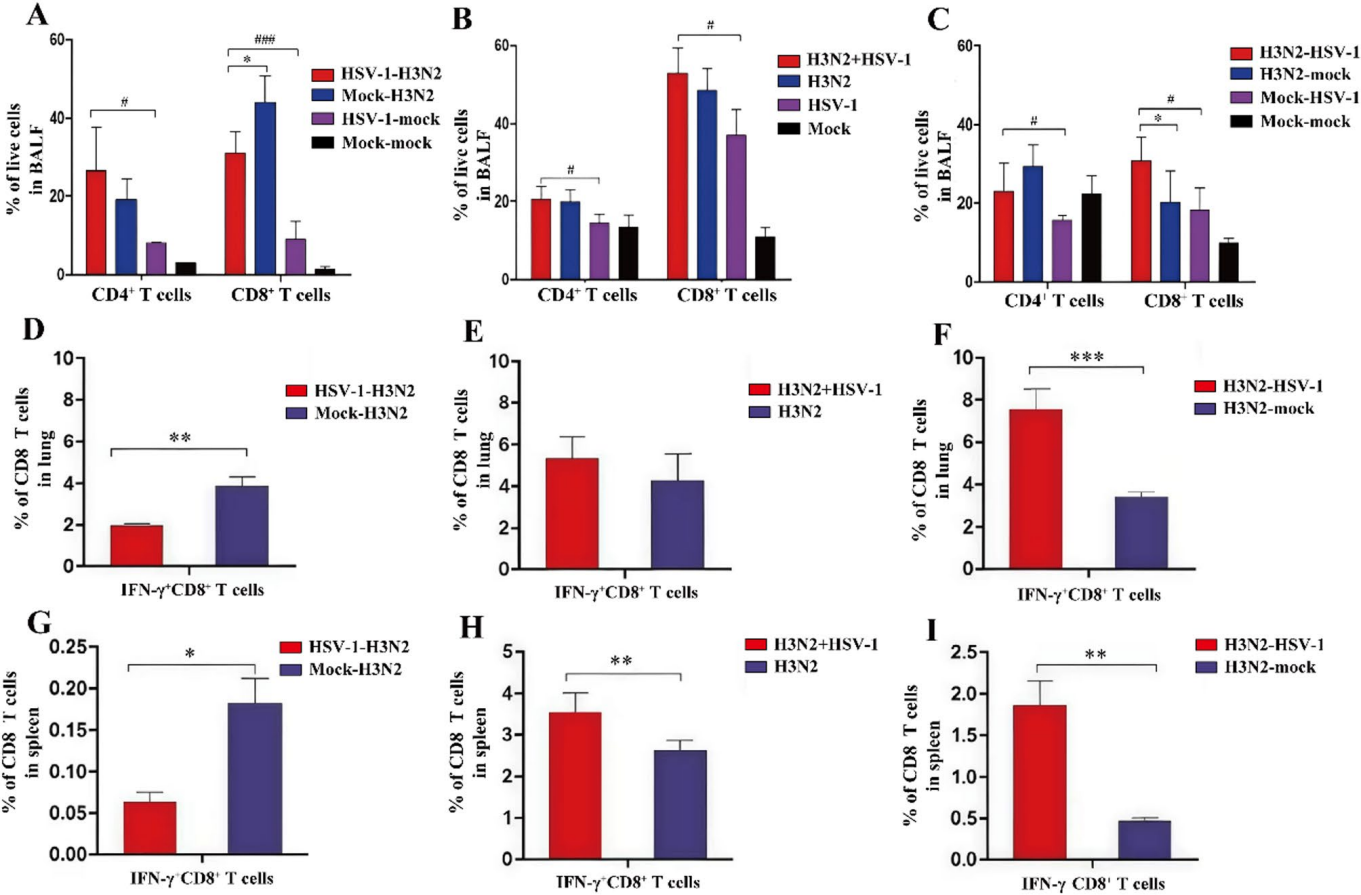
T cell response in different coinfection groups. At 8 dpi, proportions of CD4^+^ and CD8^+^ T cell subsets in BALF (**A-C**), and NP_366−374_-specific IFN-γ^+^CD8^+^ T cells in the lungs (**D-F**) and spleens (**G-I**) were measured. Sequential HSV-1-H3N2 group: (**A**), (**D**), (**G**); simultaneous H3N2 + HSV-1 group: (**B**), (**E**), (**H**); sequential H3N2-HSV-1 group: (**C**), (**F**), (**I**). Data are shown as mean ± SD. Significant differences between coinfection models and H3N2 monoinfection controls are marked by asterisk (^*^), and between coinfection models and HSV-1 monoinfection controls by hash (^#^). (^*/#^, *P* < 0.05; ^**/##^, *P* < 0.01; ^***/###^, *P* < 0.001)

Next, given the central role of adaptive immunity in antiviral defense, we analyzed T cell subsets in BALF at 8 dpi. Compared to H3N2 monoinfection controls, the HSV-1-H3N2 group exhibited significantly reduced proportions of CD8^+^ T cells (Fig. 6A), while the H3N2 + HSV-1 group showed comparable levels (Fig. 6B). In contrast, the H3N2-HSV-1 group had significantly higher levels of CD8^+^ T cell (Fig. 6C). Compared to HSV-1 monoinfection controls, all three H3N2 and HSV-1 coinfection models displayed increased proportions of CD4^+^ and CD8^+^ T cells.

To further evaluate the influenza-specific T cell response, NP_366−374_-specific IFN-γ^+^CD8^+^ T cells were measured in the lungs (Fig. 6D and F) and spleens (Fig. 6G and I) at 8 dpi. Compared to the H3N2 monoinfection controls, the HSV-1-H3N2 group displayed reduced proportions of IFN-γ^+^CD8^+^ T cell in both the lung (Fig. 6D) and spleen (Fig. 6G). In contrast, the H3N2 + HSV-1 group showed increased levels of IFN-γ^+^CD8^+^ T cells in the spleen (Fig. 6H), while the H3N2-HSV-1 group had increased levels in both the lung (Fig. 6F) and spleen (Fig. 6I).

**Fig. 7:**
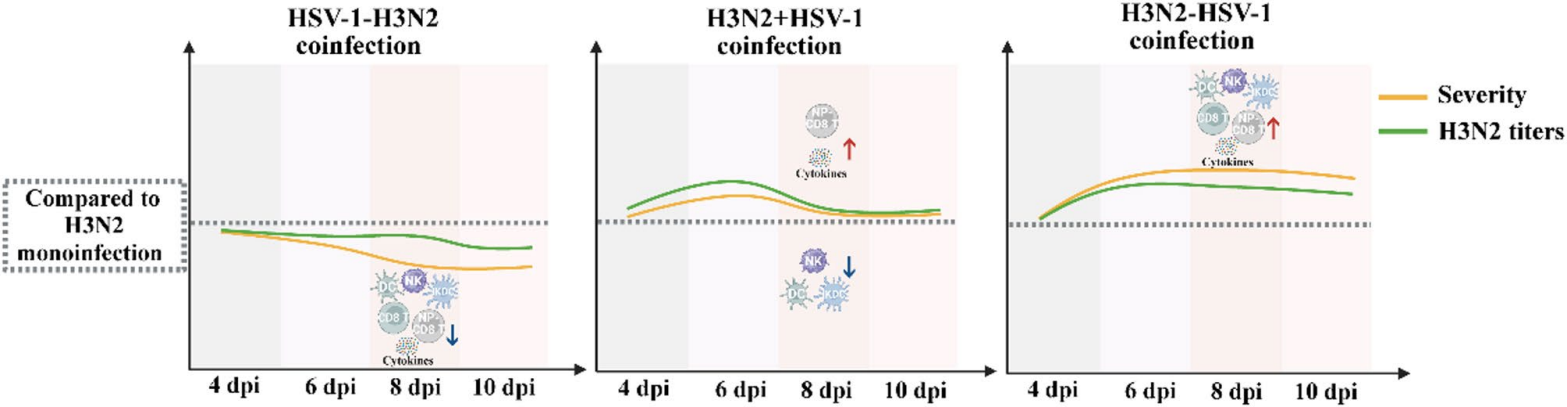
Summary of the differences in disease severity, H3N2 titers, and immune cell profiles between each of the three coinfection models and H3N2 monoinfection. Disease severity is assessed by weight loss, mortality, lung index, and histopathological analysis

These findings revealed distinct immune cell profiles driven by different orders of infection (Fig. 7). Compared to H3N2 monoinfection controls, HSV-1-H3N2 coinfection attenuated NK cells, pDCs, IKDCs, CD8^+^ T cells, and influenza-specific T cell responses. In contrast, H3N2 + HSV-1 coinfection suppressed innate immune cells but enhanced influenza-specific T cell activity, while H3N2-HSV-1 coinfection induced the expansion of NK cells, pDCs, IKDCs, CD8^+^ T cells, and influenza-specific T cells.

## Discussion

In recent years, increasing reports of HSV-1 and respiratory virus coinfections in patients with severe pneumonia have highlighted the urgent need to investigate the underlying pathogenesis [12, 15, 18, 19]. Here, we developed mouse models of coinfection with HSV-1 and influenza, which is one of the most common respiratory viruses. We found that different orders of infection led to completely opposite disease outcomes. By comparing three distinct coinfection models—sequential HSV-1-H3N2, simultaneous H3N2 + HSV-1, and sequential H3N2-HSV-1—we demonstrate that the order of infection significantly modulates viral replication, immune responses, and disease severity (Fig. 7). These results underscore the complexity of host-pathogen interactions during coinfection and provide insights into the mechanisms underlying divergent clinical outcomes.

HSV-1 is frequently detected in respiratory specimens, especially in patients requiring mechanical ventilation, which suggests that HSV-1 may act as a co-pathogen, contributing to the severity of pulmonary infections rather than being the primary cause [11, 16]. This observation aligns with our findings that HSV-1 monoinfection leads to mild disease. Therefore, we will mainly discuss on how the order of coinfection influences disease severity compared to H3N2 monoinfection.

Given the high global prevalence of latent HSV-1 infection (approximately 67%) [20], most clinical coinfections with IAV likely involve HSV-1 reactivation rather than primary infection, unlike our naive mouse model designed to isolate the effects of infection order on acute pathogenesis. The impact of HSV-1 and respiratory virus coinfection on mortality remains controversial [13, 21], as disease severity may be a cause or consequence of HSV-1 reactivation [11], which depends on the order of infection. However, determining the order of infection is challenging in clinical settings; simultaneous detection of HSV-1 and IAV confirms coinfection, yet the order of infection remains untraceable. To address this uncertainty and explore the mechanistic role of infection order, we developed a controlled mouse model to dissect the immune dynamics of HSV-1 and IAV coinfection.

We observed that the HSV-1-H3N2 group exhibited reduced H3N2 viral titers, milder lung injury, and no mortality. Likewise, in a previous study on IAV and rhinovirus (RV) coinfection, prior RV infection accelerated influenza virus clearance and attenuated disease severity, mediated by RV-induced IFN response [22]. Similarly, in a mouse model of murine gammaherpesvirus and influenza coinfection, prior herpesvirus infection followed by acute influenza infection enhanced the expansion of CD8^+^ T cells and significantly reduced influenza viral load, while also alleviating lung injury and mortality [23]. This finding supports our results in the HSV-1-H3N2 model, where prior HSV-1 infection helps control H3N2 viral replication and mitigate disease severity, suggesting that reactivation of HSV-1 (e.g., due to stress or immunosuppression) before IAV infection potentially attenuates H3N2-driven inflammation. This scenario is plausible in immunocompromised individuals [24], such as transplant recipients or HIV patients. However, in these patients with underlying immune dysfunction, even mild inflammation may lead to severe clinical consequences, underscoring the complexity of coinfection outcomes.

In contrast, the H3N2 + HSV-1 and H3N2-HSV-1 coinfection groups exhibited robust H3N2 viral replication, severe lung damage, and higher mortality. Such exacerbation suggests that, simultaneous or subsequent coinfection with HSV-1 could enhance viral replication and lung injury induced by influenza virus. The significantly enhanced replication of H3N2 virus during simultaneous H3N2 + HSV-1 coinfection suggests a potential synergistic effect, where HSV-1 potentially promotes the propagation of influenza virus. This was likely due to HSV-1 virus’s ability to rapidly inhibit type I IFN response via its encoding protein ICP27 [25]. Since IFNs are known to significantly suppress influenza propagation, the enhanced influenza replication observed in the H3N2 + HSV-1 group may result from an inefficient early antiviral response [26].

In clinical cases where IAV and HSV-1 are simultaneously detected, it is challenging to distinguish between concurrent and sequential coinfection. On the other hand, sequential IAV and HSV-1 coinfection may occur when IAV-induced immune dysregulation triggers HSV-1 reactivation, as HSV bronchopneumonitis was reported in 31% of severe influenza cases in ICU settings [13]. As observed in clinical research, secondary HSV-1 coinfection was more common in immunocompetent patients when primary pulmonary infection was severe and required mechanical ventilation [9, 13]. In these patients, the latent HSV-1 viruses in ganglia were reactivated and established pulmonary coinfection. The sequential H3N2-HSV-1 coinfection model simulated this secondary HSV-1 infection dynamics in the lung. Although its clinical impact was inconclusive [11], our findings on the increased severity of H3N2-HSV-1 coinfection support that, secondary HSV-1 infection induced more pronounced lung injury. Because primary influenza virus infection can rapidly damage the blood-gas barrier [27], then more cells are predisposed to subsequent HSV-1 infection. Secondary HSV-1 infection imposed additional stress on lungs that were already compromised by influenza-induced injury.

Furthermore, the divergent outcomes of coinfection are likely driven by differential modulation of both innate and adaptive immune responses, depending on the order of infection. In the HSV-1-H3N2 group, prior HSV-1 infection appeared to suppress the proliferation of innate immune cells induced by the subsequent H3N2 infection, including NK cells, pDCs, and IKDCs. This may allow for a more controlled immune response to H3N2 and minimize excessive pulmonary damage [28]. Both reduced innate immune cell levels and H3N2 replication led to lowered influenza-specific T cell response. The decreased IFN-γ^+^CD8^+^ T cell proportions could explain the lower IFN-γ level in BALF. In contrast, both H3N2 + HSV-1 and H3N2-HSV-1 coinfections induced a more intense influenza-specific CD8^+^ T cell response, leading to a significant increase in IFN-γ. Although in the H3N2 + HSV-1 group, NP-specific CD8 + T cells were only significantly increased in the spleen, splenic IAV-specific T cells have been reported to exhibit a similar capacity for IFN-γ secretion [29]. In these two coinfection models, enhanced H3N2 viral replication triggered a robust T cell response, and the overactive cytotoxic CD8^+^ T cell response caused extensive pulmonary parenchymal cell death and severe lung injury, leading to immunopathology [30, 31]. Overall, consistent with a previous study on Semliki Forest virus and influenza virus coinfection, our data support that prior infection with unrelated viruses can influence the magnitude and function of influenza-specific CD8^+^ T cell responses [32]. Moreover, the aberrant proliferation of NK, pDC and IKDC contributed to the heightened pro-inflammatory response in H3N2-HSV-1 coinfection [33, 34]. During H3N2 + HSV-1 coinfection, the pulmonary NK cell proportions were reduced compared to H3N2 monoinfection, a phenomenon also observed in a fatal case of influenza and RSV coinfection in an infant [35], potentially due to apoptosis, though further investigation is required.

While our study elucidates the immunopathological consequences of untreated HSV-1 and H3N2 coinfections, the potential for antiviral therapies to modulate these outcomes warrants consideration. Acyclovir, a cornerstone treatment for HSV-1 infections, inhibits viral DNA synthesis and has demonstrated prophylactic efficacy in preventing lower respiratory HSV-1 involvement in ARDS patients, thereby reducing the risk of bronchopneumonitis [36]. In mechanically ventilated patients with high HSV loads and refractory pneumonia, acyclovir administration has been linked to improved survival and enhanced lung function [37], highlighting its capacity to alleviate virus-driven tissue damage. Given the exacerbated inflammation observed in our simultaneous and H3N2-HSV-1 groups, acyclovir could potentially attenuate IAV-exacerbated pathology by curbing HSV-1 replication and associated cytokine dysregulation. However, further studies are needed to evaluate its impact in coinfection scenarios.

While our in vivo model captures complex host-virus interactions, in vitro studies provide complementary insights into HSV-1 and IAV mechanisms, though direct coinfection data are limited. For instance, HSV-1 infection induces novel circular and linear splicing of the long NEAT1 isoform in human fibroblasts, a process also observed with IAV in epithelial cells, suggesting shared alterations in host RNA processing that could facilitate viral synergy by impairing host antiviral responses [38]. Additionally, HSV-1 blocks virus-induced IFN-β production via inhibition of IRF-3 nuclear accumulation in endometrial cells, potentially enhancing IAV replication by suppressing type I IFN pathways [39]. Superinfection exclusion (SIE), a common form of viral interference mediated by HSV-1, involves HSV-1 glycoprotein D downregulating nectin-1 to limit homologous viral entry [40, 41]. This mechanism is less relevant for heterologous viruses like IAV. In our study, elevated H3N2 viral loads in simultaneous and H3N2-HSV-1 coinfections indicate that immune-mediated effects override potential SIE, consistent with our findings that prior HSV-1 infection attenuates disease severity through controlled immune responses, while simultaneous or H3N2-preceding HSV-1 coinfections exacerbate immunopathology via IFN-inhibition-associated IAV replication and overactive T cell proliferation. Future in vitro coinfection studies could further elucidate direct viral interactions to complement our findings.

While this study provides valuable insights, it has certain limitations. First, we used a mouse model, which may not fully replicate the complexity of human immune responses during coinfection. Besides, the study focused on acute infection, and the long-term consequences of HSV- and influenza coinfection remain to be explored. Future studies should investigate the role of additional immune cell populations, such as regulatory T cells and B cells, as well as the impact of coinfection on tissue repair and recovery.

## Conclusion

In summary, this study demonstrates that the order of infection during influenza and HSV-1 coinfection critically shapes disease outcomes by modulating immune responses. Sequential HSV-1-H3N2 coinfection confers protection by suppressing excessive inflammation and promoting viral clearance, while simultaneous and H3N2-preceding coinfection exacerbate disease severity through enhanced inflammation and dysregulated antiviral immunity. These findings highlight the importance of considering infection timing and immune modulation strategies in future studies, as well as in the clinical management of viral coinfections.

## Data Availability

The datasets used and/or analysed during the current study are available from the corresponding author on reasonable request.

## Abbreviations

IAV: Influenza A virus
HSV-1: Herpes simplex virus type 1
PFU: Plaque forming unit
Dpi: Days post infection
H&E: Hematoxylin and eosin
BALF: Bronchoalveolar lavage fluid
NP: Nucleoprotein
IFN-γ: Interferon-gamma
TNF-α: Tumor necrosis factor-alpha
IP-10: Interferon-gamma-induced protein 10
MCP-1: Monocyte chemoattractant protein-1
IL-10: Interleukin-10
IL-6: Interleukin-6
NK: Natural killer
pDC: Plasmacytoid dendritic cells
IKDC: Interferon-producing killer dendritic cells
